# Supplementary material for: Comparing modelling approaches for the estimation of government intervention effects in COVID-19: Impact of voluntary behavior changes
Source: PLoS One. 2023 Feb 15;18(2):e0276906. doi: 10.1371/journal.pone.0276906 (PMC9931149; doi:10.1371/journal.pone.0276906)
Supplement: S1 File — (DOCX) [file pone.0276906.s001.docx]

Supplementary Methods for

Comparing modelling approaches for the estimation of government intervention effects in COVID-19: Impact of voluntary behavior changes

Lun Liu, Zhu Zhang, Hui Wang, Shenhao Wang, Shengsheng Zhuang, Jishan Duan

Correspondence to: [wh-sa@mail.tsinghua.edu.cn](mailto:wh-sa@mail.tsinghua.edu.cn)

Supplementary Methods

1 Testing common trend assumption: Two-way fixed effect estimator

To acquire reliable estimates from difference-in-difference analysis, the data need to meet the assumption of parallel trend, meaning that the outcome of interest should move in parallel trend in all units, absence of intervention. The test is implemented with an event study design, by adding terms of pre-intervention periods to the basic two-way fixed-effect model as placebo tests. If the parallel trend assumption is satisfied, there should be no significant difference between the outcomes in treatment and control groups, so that the coefficients for pre-intervention variables should be statistically insignificant. The testing model is specified as follows


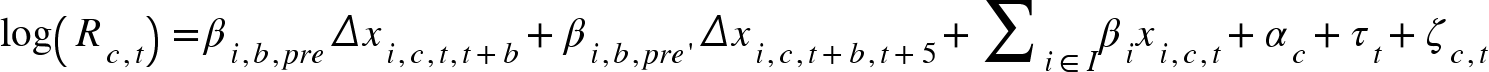
 (13)

where *Δx_i,c,t,t+b_* (*b* ≤ 5) denotes the change in the status of intervention *i* in county *c* from day *t* to day *t+b* and the same applies to *Δx_i,c,t+b,t+5_*. Correspondingly, *β_i,b,pre_* denotes the estimate of pre-trend *b* days before a change, which is the coefficient of concern; *β_i,b,pre’_* is to control for the period *b* to 5 days before a change (if *b*=5, then the estimate is NA), so that all analyses use the period more than 5 days before a change as the reference. We test the pre-trend in 1 to 5 days before an intervention, since interventions tend to be quick decisions. The rest of the notations are the same as in Eq. 8.

We estimate *β_i,b,pre_* for all intervention *i* and for *b*∈{1, 2, 3, 4, 5}. The estimates of *β_i,b,pre_* are shown in S3 Table. The pre-trends of all the interventions are statistically insignificant in most of the pre-periods, suggesting that the epidemic courses in control and treatment groups generally moved in parallel and the intervention effect estimates in the main texts are reliable.

**2 Testing common trend assumption: Robust estimator**

For the robust estimator, the common trend assumption is also examined through placebo tests that estimate the impact of being *k* days before an intervention change. This is implemented by modifying Eq. 9-11 as follows.


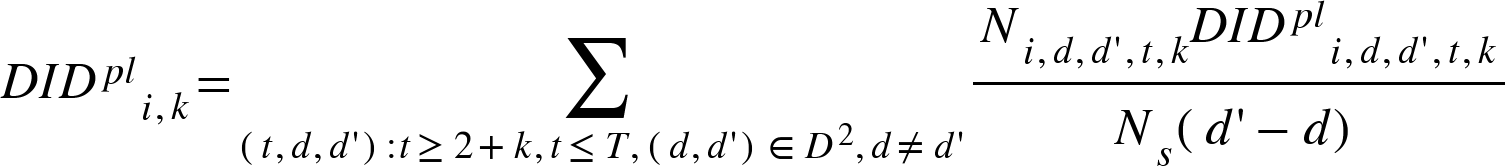
 (14)


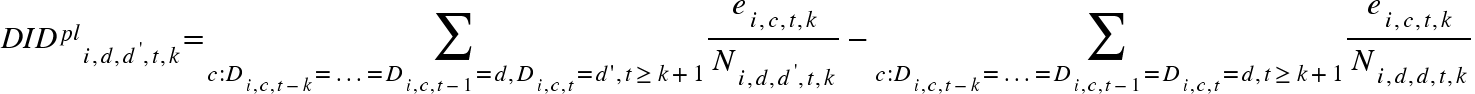
 (15)


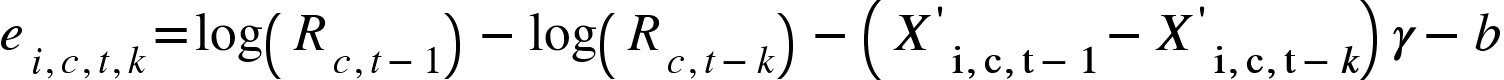
 (16)

We conduct the test for all intervention *i* and for *k*∈{1, 2, 3, 4, 5}. The estimates of *DID^pl^_i,k_* are shown in S4 Table. All estimates are statistically insignificant, suggesting that the intervention effect estimates in the main texts should be reliable.
