# Supplementary material for: Comparing modelling approaches for the estimation of government intervention effects in COVID-19: Impact of voluntary behavior changes
Source: PLoS One. 2023 Feb 15;18(2):e0276906. doi: 10.1371/journal.pone.0276906 (PMC9931149; doi:10.1371/journal.pone.0276906)
Supplement: S1 Table — Sample and results are provided if the study takes Rt as the outcome of interest, which are comparable to our analysis. (DOCX) [file pone.0276906.s002.docx]

**S1 Table. Methodological summary of studies on COVID-19 intervention effect.** Sample and results are provided if the study takes *R_t_* as the outcome of interest, which are comparable to our analysis.

| **Study** | **Method** | **Sample** | **Result (with 95% CI)** |
| --- | --- | --- | --- |
| Banholzer et al., 2021 [16] | Bayesian model |  |  |
| Bo et al., 2021 [20] | Linear mixed regression | 235 cities from 10 countries and 180 countries, January 23 to April 13 2020 | Reduction in *R_t_*:  Wearing mask 15% (9-22%)  Quarantine 11% (9-14%)  Social distancing 43% (42-44%)  Traffic restriction 9% (7-11%) |
| Brauner et al., 2020 [2] | Bayesian model | 41 countries, January to May 2020 | Reduction in *R_t_*:  Gatherings limited to 1000 people or less 23% (0-40%)  Gatherings limited to 100 people or less 34% (12-52%)  Gatherings limited to 10 people or less 42% (17-60%)  Closing some businesses 18% (−8-40%)  Closing most businesses 27% (−3-49%)  Closing schools and universities 38% (16-54%)  Issuing stay-at-home orders 13% (−5-31%) |
| Chaudhry et al., 2020 [21] | Negative binomial regression |  |  |
| Cho, 2020 [13] | Synthetic control |  |  |
| Courtemanche et al., 2020 [11] | Difference-in-Difference (event study) |  |  |
| Dave et al., 2021 [10] | Difference-in-Difference |  |  |
| Dreher et al., 2021 [22] | Linear and logistic regression |  |  |
| Esra et al., 2020 [17] | Bayesian model | 26 countries and 34 US states, up to May 2020 | Reduction in *R_t_*:  Household confinement 23% (18-27%)  Limits on gatherings 10% (1-18%)  School closures 12% (5-19%)  Mask policies 17% (6-28%) |
| Flaxman et al., 2020 [3] | Bayesian model | 11 European countries, up to May 2020 | Reduction in *R_t_*:  Lockdown 81% (75-87%)  Public event bans, school closure  self-isolation and social distancing encouraged: numbers not given in text, but close to zero |
| Fowler et al., 2020 [8] | Difference-in-Difference |  |  |
| Haug et al., 2020 [5] | Case-control  LASSO regression  Random forests  Transformers | 226 countries, March-April 2020 | Reduction in *R_t_*:  Banning small gatherings 0.22-0.35  Banning mass gatherings 0.13-0.33  Educational institution closure 0.15-0.21  Border restriction 0.057-0.23  Individual movement restriction 0.08-0.13  National lockdown 0.008-0.14 |
| Hsiang et al., 2020 [4] | Two-way fixed effect model |  |  |
| Islam et al., 2020 [14] | Interrupted time series analysis |  |  |
| Koh, Naing & Wong, 2020 [23] | Linear regression | 142 countries, two weeks following the 100^th^ reported case | Reduction in *R_t_*:  Border closure 24% (−1-50%)  Stay-at-home recommendation 45% (7-82%)  Partial lockdown 38% (4-72%)  Complete lockdown 32% (9-55%) |
| Leffler, et al., 2020 [24] | Linear regression |  |  |
| Olney et al., 2021 [18] | Bayesian model |  |  |
| Piovani et al., 2021 [25] | Negative binomial regression |  |  |
| Pozo-Martin et al., 2021 [26] | Mixed effect linear regression + Bayesian estimation |  |  |
| Sharma et al., 2021 [19] | Bayesian model | 114 regions in 7 European countries, August 2020 to January 2021 | Reduction in *R_t_*:  Gastronomy closures 12% (8-17%)  Night club closures 12% (8-17%)  Retail and contact service closures 12% (7-18%)  Leisure and entertainment venue closures 3% (−1-10%)  Banning all gatherings 26% (18-32%)  Educational institution closures 7% (4-10%) |
| Siedner et al., 2020 [37] | Mixed effect linear regression (similar to two-way fixed effect model) |  |  |
| Singh et al., 2021 [6] | Difference-in-difference |  |  |
| Wibbens, Koo & McGahan, 2020 [38] | Bayesian model |  |  |
