# Supplementary material for: Comparing modelling approaches for the estimation of government intervention effects in COVID-19: Impact of voluntary behavior changes
Source: PLoS One. 2023 Feb 15;18(2):e0276906. doi: 10.1371/journal.pone.0276906 (PMC9931149; doi:10.1371/journal.pone.0276906)
Supplement: S2 Table — (DOCX) [file pone.0276906.s003.docx]

**S2 Table. Results of models on intervention status and residents’ mobility**

| **Variable** | **Model 1** | **Model 2** |
| --- | --- | --- |
| Stay-at-home order | 5.393***  (0.096) | 1.387***  (0.051) |
| School closure | -0.372**  (-2.993) | -0.054  (0.116) |
| Childcare closure | 1.192***  (0.123) | 2.845***  (0.086) |
| Non-essential retail closure | 1.196***  (0.097) | 0.719***  (0.065) |
| Small-size gathering ban | 0.821***  (0.053) | -1.067***  (0.039) |
| Large-size gathering ban | 1.946***  (0.091) | 2.503***  (0.062) |
| Intercept | 6.836***  (0.096) | 1.816***  (0.194) |
| Day | No | Yes |
| N | 66037 | 66037 |
| Adj. R^2^ | 0.210 | 0.647 |

* *p*<0.05, ** *p*<0.01, *** *p*<0.001
