# Supplementary material for: Comparing modelling approaches for the estimation of government intervention effects in COVID-19: Impact of voluntary behavior changes
Source: PLoS One. 2023 Feb 15;18(2):e0276906. doi: 10.1371/journal.pone.0276906 (PMC9931149; doi:10.1371/journal.pone.0276906)
Supplement: S3 Table — (DOCX) [file pone.0276906.s004.docx]

**S3 Table. Pre-trend test for the two-way fixed effect estimator**

| **Intervention** | **1 day** | **2 days** | **3 days** | **4 days** | **5 days** |
| --- | --- | --- | --- | --- | --- |
| Stay-at-home | -0.029  (0.0191) | -0.0265  (0.02) | -0.0269  (0.0208) | -0.024  (0.0216) | -0.0212  (0.0224) |
| School | -0.0339  (0.0631) | -0.0369  (0.0666) | -0.0253  (0.0713) | -0.00537  (0.0809) | 0.0125  (0.0917) |
| Childcare | -0.0637  (0.041) | -0.085*  (0.0417) | -0.0907*  (0.0438) | -0.0823  (0.0465) | -0.085  (0.0467) |
| Retail | -0.0148  (0.029) | -0.0222  (0.0299) | -0.0249  (0.0312) | -0.0241  (0.0328) | -0.0273  (0.0338) |
| Small gathering | -0.0517*  (0.0249) | -0.0448  (0.0254) | -0.0332  (0.0261) | -0.0261  (0.0267) | -0.021  (0.0267) |
| Large gathering | -0.0458  (0.0426) | -0.0208  (0.0441) | 0.00747  (0.0467) | 0.0166  (0.049) | 0.0198  (0.0509) |

* *p*<0.05, ** *p*<0.01, *** *p*<0.001
