# Supplementary material for: Comparing modelling approaches for the estimation of government intervention effects in COVID-19: Impact of voluntary behavior changes
Source: PLoS One. 2023 Feb 15;18(2):e0276906. doi: 10.1371/journal.pone.0276906 (PMC9931149; doi:10.1371/journal.pone.0276906)
Supplement: S4 Table — (DOCX) [file pone.0276906.s005.docx]

**S4 Table. Pre-trend test for the robust difference-in-difference estimator**

| **Intervention** | **1 day** | **2 days** | **3 days** | **4 days** | **5 days** |
| --- | --- | --- | --- | --- | --- |
| Stay-at-home | -0.0103  (0.00734) | 0.0148  (0.0153) | -0.00226  (0.0121) | 0.00863  (0.0169) | 0.00136  (0.0285) |
| School | -0.00648  (0.0242) | -0.03  (0.0323) | -0.0564  (0.0595) | 0.0149  (0.0804) | 0.0694  (0.113) |
| Childcare | 0.0374  (0.0383) | 0.0513  (0.0539) | -0.0153  (0.0438) | -0.0371  (0.0484) | 0.0161  (0.0364) |
| Retail | 0.0256  (0.0304) | 0.00314  (0.0253) | 0.00696  (0.0333) | 0.0179  (0.0311) | 0.0212  (0.0436) |
| Small gathering | -0.0144  (0.0241) | -0.0278  (0.0234) | -0.00071  (0.0269) | -0.0196  (0.0183) | -0.0353  (0.0206) |
| Large gathering | 0.00662  (0.0483) | -0.031  (0.0462) | -0.00597  (0.0618) | -0.0633  (0.0595) | -0.0103  (0.0565) |

* *p*<0.05, ** *p*<0.01, *** *p*<0.001
